# Soybean Meal-Based Wood Adhesive Enhanced by Phenol Hydroxymethylated Tannin Oligomer for Exterior Use

**DOI:** 10.3390/polym12040758

**Published:** 2020-03-31

**Authors:** Mingsong Chen, Yi Zhang, Yue Li, Sheldon Q. Shi, Jianzhang Li, Qiang Gao, Hongwu Guo

**Affiliations:** 1 MOE Key Laboratory of Wooden Material Science and Application, Beijing Forestry University, No.35 Tsinghua East Road, Beijing 100083, China; chenmingsong@bjfu.edu.cn (M.C.); luckyyizhang@163.com (Y.Z.); liyue0215@bjfu.edu.cn (Y.L.); lijzh@bjfu.edu.cn (J.L.); 2 College of Engineering Department of Mechanical and Energy Engineering, University of North Texas, 3940 North Elm street, Suite F101P, Denton, TX 76207-7102, USA; Sheldon.shi@unt.edu

**Keywords:** soybean meal adhesive, tannin, bond strength, plywood, exterior use

## Abstract

Bio-based adhesives have low water resistance and they are less durable than synthetic adhesives, which limits their exterior applications. In this study, a bio adhesive was developed from soybean meal and larch tannin that was designed for exterior use. Phenol hydroxymethylated tannin oligomer (PHTO) was synthesized and then mixed with soybean meal flour in order to obtain a soybean meal-based adhesive (SPA). The results showed that the moisture absorption rate, residual rate, and solid content of SPA with 10 wt % PHTO (mass ratio with respect to the entire adhesive) were improved by 22.8%, 11.6%, and 6.8%, respectively, as compared with that of pure SPA. The wet shear strength of plywood with SPA with 10 wt % PHTO (boiling in 100 °C water for 3 h) was 1.04 MPa when compared with 0 MPa of pure SPA. This met the bond strength requirement of exterior-use plywood (GB/T 9846.3-2004). This improved adhesive performance was mainly due to the formation of a crosslinked structure between the PHTO and the protein and also PHTO self-crosslinking. The formaldehyde emission of the resulting plywood was the same as that of solid wood. The PHTO-modified SPA can potentially extend the applications of SPAs from interior to exterior plywood.

## 1. Introduction

Urea-formaldehyde (UF) resins and its modified productions dominate the plywood manufacturing industry. However, these resins tend to cause environmental pollution and some of the raw materials are derived from non-renewable resources [1,2]. Soy protein, which is a by-product of the soybean oil processing industry, is a renewable natural resource, making it an ecofriendly, inexpensive, and readily available raw material for bio-based wood adhesives. However, the use of soy protein has been hindered by its poor water resistance [3,4], because the bond strength of soybean meal-based adhesive (SPA) primarily depends on the physical interlocking of protein molecules and hydrogen bonds, which are easily disrupted by moisture [5,6]. Numerous recent studies have explored various strategies to improve the water-resistance of SPAs, including denaturation [7], grafting [8], and crosslinking [9]. Forming a crosslinked network is the simplest and most effective method for improving water resistance and adhesive performance [10]. Epoxides and polyisocyanates cross-linkers have been employed to enhance the adhesive performance of SPAs [11], and the resultant panels met the requirements for interior-use panels. However, it is still difficult to fabricate bio-based adhesives for exterior-use panels. In addition, these cross-linkers are completely dependent on costly non-renewable raw materials, which, in turn, make the SPAs overly reliant on such materials. Thus, we propose the development of an exterior-use wood adhesive derived from soybean meal and a crosslinker.

Phenol-formaldehyde (PF) resins are exterior-use adhesives that have excellent water resistance and durability due to the presence of the rigid benzene moieties. Larch (*Larix gmelinii*) tannin (LT) has been a focal point in plant-based materials research due to its highly condensed tannin content and high water-solubility. LTs are polymeric phenol compounds containing flavon-3-ol repeat units, which gives them the potential for use as outdoor phenol-formaldehyde resins. Flavonoids and catechins are important LT structural components that have the same skeleton structure—a carbon skeleton that contains two phenyl rings (A and B) and a heterocyclic ring (C) [12]. The polycondensation reaction is the most well-known reaction that is associated with the A-ring [13]. Many studies have shown that tannin easily reacts with the aldehyde group due to its polyphenolic nature [14,15], which can be used to replace a portion of phenol to develop an LT-based phenol-formaldehyde resin for exterior-use plywood. LT is also highly reactive because of its unique structure and can, therefore, be used to synthesize SPA cross-linkers and be used to introduce a polymeric phenol compound into a SPA formulation in order to improve the water resistance and durability of the resultant adhesive.

In this study, LT was initially treated with small amounts of phenol and formaldehyde to develop a phenol hydroxymethylated tannin oligomer (PHTO), which was then used as a bio-based cross-linker to modify and fabricate a high-performance SPA. Attenuated total reflectance-Fourier transform infrared (ATR-FTIR) spectroscopy and Thermogravimetric Analysis (TGA) characterized the PHTO. The functional groups, thermal behavior, hydrolytic stability, viscosity, solid content, and moisture absorption rate of the adhesive were measured. In addition, three-ply poplar plywood samples that were bonded by the resulting adhesive were prepared to investigate the bonding strength and formaldehyde emission.

## 2. Materials and Methods

### 2.1. Materials

Soybean meal (SM, 46.8% *w*/*w*) was purchased from Xiangchi Grain and Oil Co., Ltd. (Zibo, China). Larch tannin (LT, 60.1% *w*/*w*) was obtained from Tian’guan Biotech Co. (Nanyang, China). Pure phenol, formaldehyde solution (37 wt %), and sodium hydroxide (NaOH) were analytical-grade reagents and they were obtained from Beijing Chemical Reagents Co., Ltd. (Beijing, China). The poplar veneers (moisture content of 8.0%, dimensions of 400 mm × 400 mm × 1.5 mm) were purchased from Wen’an Plywood Ltd. (Hebei, China).

### 2.2. Preparation of Phenol Hydroxymethylated Tannin Oligomer (PHTO)

The raw commercial larch tannin (LT) powder with an LT content of 60.1 wt % was used to develop the PHTO while using the following procedure. Pure phenol (55 g) was added into a three-neck flask. The LT solution (187.5 g) with 24 wt % LT was added and then stirred at a rotation rate of 400 rpm for 15 min. A formaldehyde solution (104.4 g, formaldehyde based on a molar ratio 2.2:1 with respect to phenol) was added into the three-neck flask and then continuously stirred. The temperature was increased from 45 °C to 88 °C over a period of 45 min. The mixture was held at 88 °C for another 15 min. and was then cooled to 45 °C for another 2 h. Subsequently, a NaOH solution (4.2 g, 50 wt %) was used to catalyze the reaction and maintain a stable pH of 10.0. Finally, the sample was cooled to 25 °C and a homogeneous brown solution was obtained. Table 1 reports the final values for the primary physical properties of the synthesized PHTO.

### 2.3. Physicochemical Properties of PHTO

The physicochemical properties of PHTO include viscosity, solid content, pH, functional groups, and thermal stability. The viscosity of the PHTO sample was measured by a HAAKE RotoVisco 1 rheometer (Thermo Scientific, Waltham, MA, USA) with a 35 mm plate diameter parallel plate fixture (PP35). The solid content of the PHTO sample was determined while using an oven-drying method, in which 3 g of adhesive were oven-dried at 105 ± 2 °C until a constant weight was obtained. The solid content was determined by weighing the sample before and after drying, and then subtracting the latter value from the former. The pH of the PHTO sample was determined using an MT-5000 pH meter (Shanghai precision instrument co., LTD, Shanghai, China) [16].

ATR-FTIR spectroscopy measured the functional groups of LT and PHTO. The samples for ATR-FTIR were prepared by oven-drying the adhesives at 120 ± 2 °C until completely cured, and then milled into powder and sifted with a 200 mesh to obtain particles 0.075 mm in size. ATR-FTIR spectroscopy was conducted using a Thermo Nicolet 6700 FTIR Spectrometer (Thermo Fisher Scientific, Waltham, MA, USA) over the range of 650–4000 cm^−1^ at 4 cm^−1^ resolution [17]. 

Thermogravimetric (TG) measurements were recorded on a TGA instrument (TA Q50, Waters Company, New Castle, DE, USA). Approximately 5 ± 0.1 mg of the 0.075 mm powder samples were placed in a platinum cup and then sealed in a nitrogen environment. The samples were then scanned while heated from 30 °C to 600 °C at a 10 °C/min. heating rate. Changes in the sample weight were recorded throughout and then analyzed [18].

### 2.4. Preparation of Soybean Meal-Based Adhesives

200-mesh SM powder was ground and then prepared using a grinder. SM powder (28 g) was mixed with deionized water (72 g). The SM adhesive (adhesive 1) was prepared by stirring the mixture at 600 rpm for 15 min. to form a homogeneous system. For the PHTO-modified SPAs, various amounts of PHTO (5, 10, 15, and 20 g) were incorporated into the pure SM adhesive and then stirred for additional 15 min. to obtain PHTO-modified SPAs, which were labeled as adhesive 2, adhesive 3, adhesive 4, and adhesive 5, respectively. 

### 2.5. Characteristics of the Adhesive Samples

#### 2.5.1. ATR-FTIR Spectroscopy 

The adhesives were oven-dried at 120 ± 2 °C for about 3 h until completely cured. The cured adhesive samples were ground to 200 mesh powder and scanned with a Thermo Nicolet 6700 FTIR Spectrometer following the same procedure outlined in Section 2.3.

#### 2.5.2. Hydrolytic Stability

Hydrolytic stability was determined via a residual rate test [19]. The adhesive samples were oven-dried at 120 ± 2 °C for about 3 h until a constant weight (*M*) was obtained. Subsequently, the samples were ground into a 100-mesh powder using a ceramic mortar. The ground powder was wrapped up using a qualitative filter paper and then soaked in distilled water. After hydrolyzing at 60 ± 2 °C for 6 h, the qualitative filter paper with the hydrolyzed sample was placed in an oven again at 105 ± 2 °C for 3 h until a constant weight (*m*) was obtained. The residual rate value was defined as *m* divided by *M* and expressed as a percentage. All of the measurements were conducted in triplicate.

#### 2.5.3. TG Analysis/Viscosity/Solid Content/pH Value Measurements

The TG, solid content, viscosity, and pH value measurement method of samples followed the same procedure that was described in Section 2.3. 

#### 2.5.4. Moisture Absorption Measurement

Moisture absorption was measured via the mass-loss test [20]. The weight of a cured adhesives (*m*_0_) was recorded every two hours at 50 °C and 80% RH until a constant weight (*m*_1_) was obtained. The moisture absorption value was calculated by the following equation:

$$\mathrm{Moisture}\;\mathrm{absorption}\;\mathrm{value}\;\left(\%\right)=\frac{m_{1}-m_{0}}{m_{0}}\times 100\%$$

#### 2.5.5. SEM Analysis

Prior to SEM analysis, liquid SPAs were oven-dried at 120 ± 2 °C for 3 h to obtain cured adhesives. Field emission SEM (Hitachi S-4800, Tokyo, Japan) was used to observe the cross-sectional morphologies of the cured adhesives [21].

### 2.6. Characteristics of Plywood Samples

The wet shear strength was evaluated according to the Chinese National Standards (GB/T 9846.3-2004). The glued specimens were fabricated by spreading 200 g·m^–2^ of glue (when considering the whole adhesive including powder, modifier and water) in a single glue line. The glued veneers were assembled and hot-pressed for 315 s at 1.0 MPa and 120 °C. The plywood samples were stored at 25 °C and 50% relative humidity (RH) for at least 24 h before being cut into a size of 100 mm × 25 mm. Six specimens with bonding zone sizes of 25 mm × 25 mm were cut out, soaked in boiling water for 3 h, and then cooled at ambient temperature for 10 min. before testing. 

The formaldehyde emission values of the plywood samples were also evaluated according to the Chinese National Standards (GB/T 9846.3-2004). Ten pieces with the dimensions of 150 mm × 50 mm were placed in a 10 L glass desiccator, which contained a dish with 300 mL of distilled water to absorb and determine formaldehyde. The desiccator was held at 25 °C and 50% RH for 24 h. The average value of the formaldehyde concentration was calculated for the three panels.

## 3. Results and Discussion

### 3.1. Physical Properties and Chemical Structure of PHTO

The characteristics of condensed tannins mainly depend on their botanical source, as mentioned in the Introduction. Condensed tannins from larch bark extracts are primarily composed of flavan-3-ols repeat units [22]. Flavonoid units in procyanidins (phloroglucinol A-ring; catechol B-ring) are typically linked to each other at C4–C8 and C4-C6. Especially, the A-ring of flavan-3-ols units rapidly and irreversibly reacts with formaldehyde [23]. 

**Figure 1:**
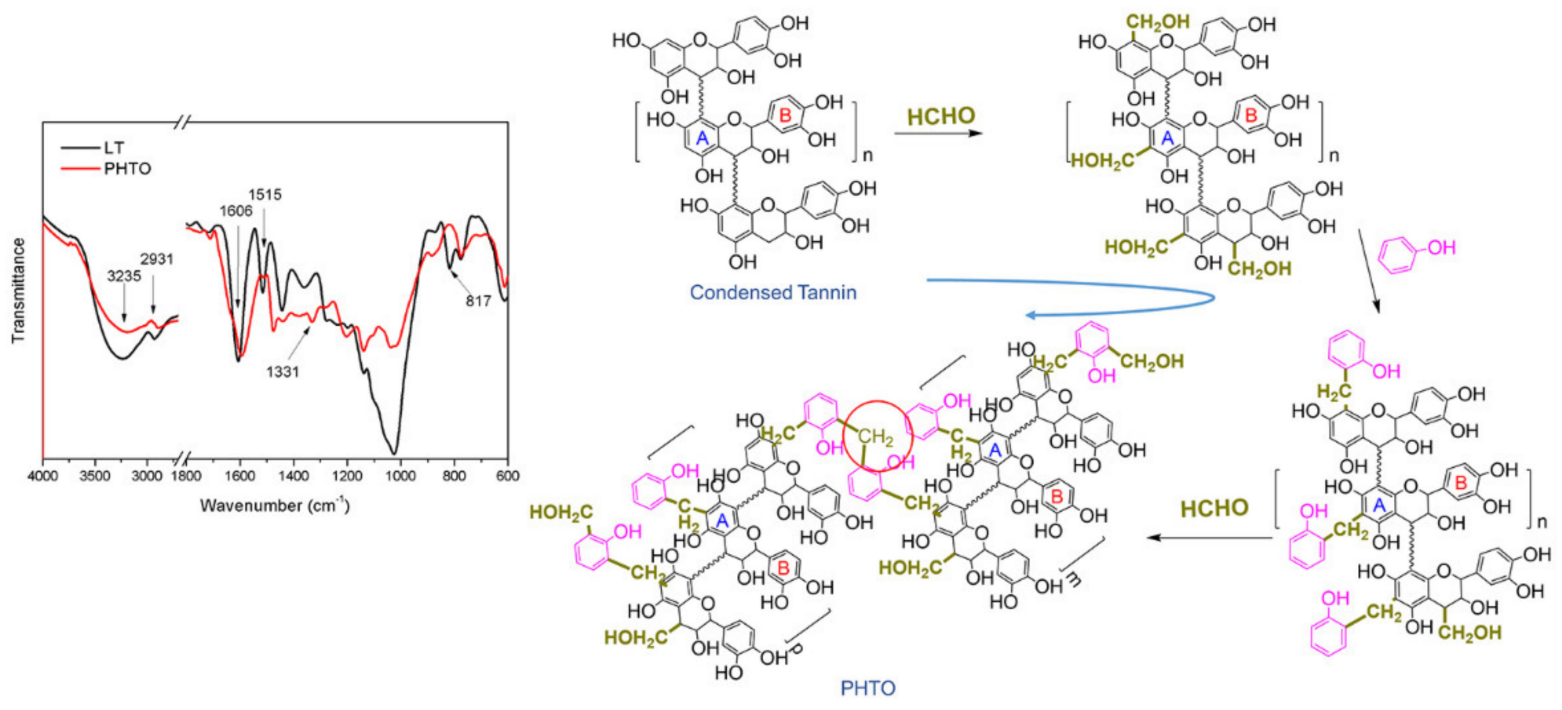
Fourier transform infrared (FTIR) spectra and the proposed reaction of larch tannin (LT) and phenolic hydroxymethylated tannin oligomer (PHTO).

FTIR spectroscopy can be used to evaluate the interactions between the LT and formaldehyde/phenol. Figure 1 presents the FTIR spectra of LT and PHTO. The broad peak in the LT spectrum near 3235 cm^−1^ is characteristic of water hydroxyl groups; the small peak at 2931 cm^−1^ is due to aromatic C–H stretching vibrations; and, the peak at 1606 cm^−1^ is due to the deformation of aromatic rings [24]. The peak at 1606 cm^−1^ appeared in the spectrum of PHTO, which indicayed that the main structure of tannin had been preserved. There were two peaks in the LT spectrum at 1515 cm^−1^ and 817 cm^−1^, which were attributed to C–H bending of the benzene ring in tannin. Compared with the LT spectrum, these two peaks disappeared in the spectrum of PHTO, indicating that the H of the benzene ring in tannin was replaced by the reaction between tannin and formaldehyde to form hydroxymethylated groups. This result was in accordance with the research of Tondi [25]. The absorption at 1331 cm^−1^ in the PHTO spectra was assigned to the C–C stretching vibration, which was attributed to the polycondensation of hydroxymethyl groups with themselves [26]. The absorption at 1040–1280cm^−1^ in the LT spectrum was assigned to the C-O stretching vibration. This band was reduced in the spectrum of PHTO, which indicated that the C-O in polymeric LT was broken during polymerization [27,28]. Figure 1 depicts the reaction mechanism of PHTO. Differential thermal gravimetry (DTG, in Figure S1) showed that two peaks in the tannin curve at 225 °C and 272 °C merged with another two peaks. New peaks appeared between 300 °C and 500 °C, which indicated phenol hydroxymethylation changed tannin’s thermal properties and produced a more thermally stable crosslinked structure. These results are consistent with the FTIR analysis. 

### 3.2. Performance Analysis of the Resultant SPAs

#### 3.2.1. Thermal Stability and FTIR Analysis

**Figure 2:**
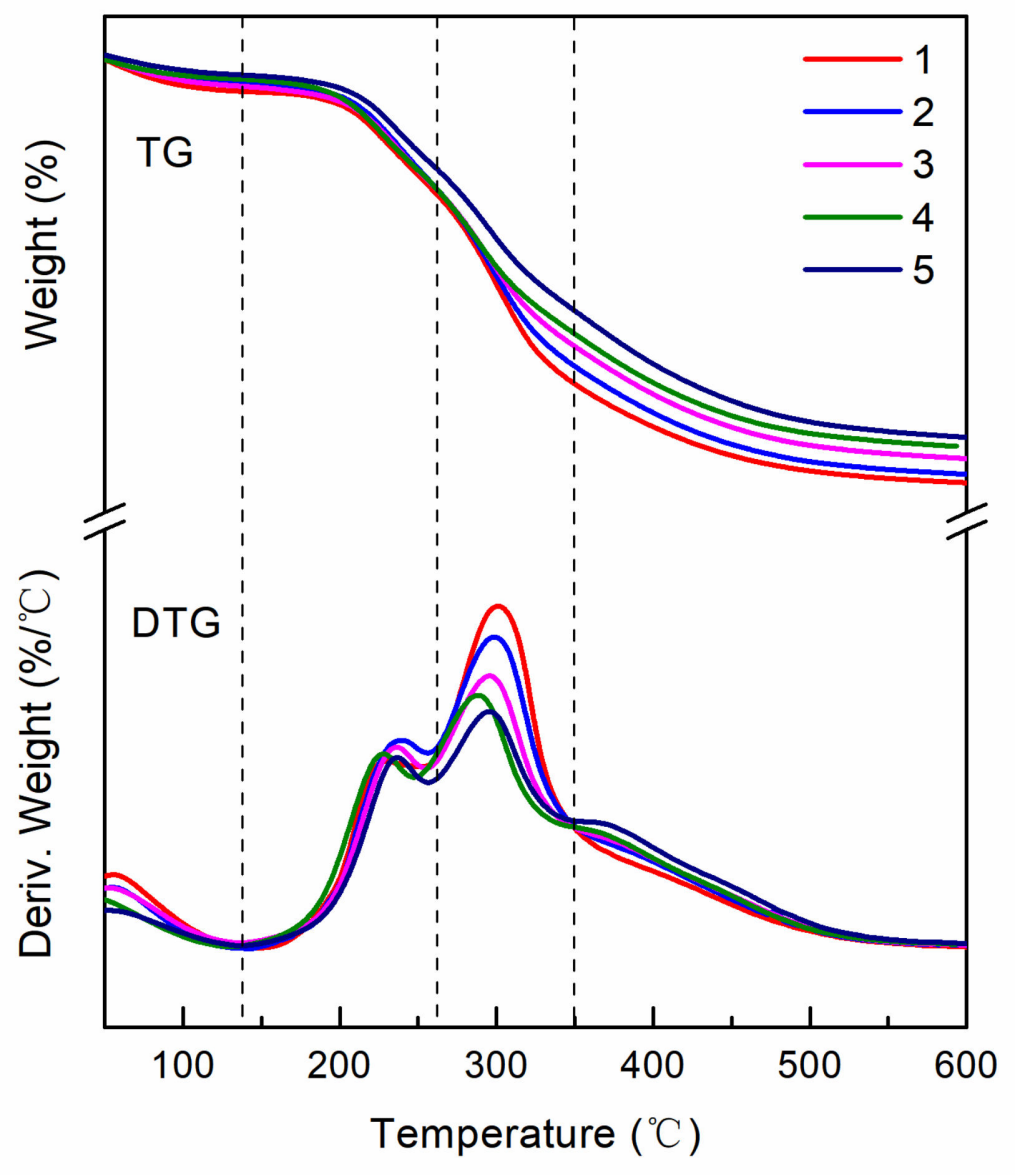
Thermogravimetric Analysis (TGA) curves and their differential curves of the different adhesives: 1 (pure SM adhesive), 2 (5 wt % PHTO/SPA), 3 (10 wt % PHTO/SPA), 4 (15 wt % PHTO/SPA), and 5 (20 wt % PHTO/SPA).

Figure 2 shows the thermal degradation of the various cured adhesive samples and it can be divided into three stages. 

Stage I occurred over the 30–140 °C temperature range, and it was primarily associated with the evaporation of residual moisture. During this stage, the SPAs showed no degradation and only a small weight loss ratio. Stage II occurred from 140–260 °C and it is the initial degradation stage. In this stage, the first weight loss occurred due to the degradation of small molecules and the rupture of unstable chemical bonds. Stage III, from 260–350 °C, was responsible for the degradation of the skeletal structure, which resulted in the thermal degradation of the cross-linked structure. Additional heating caused the breakage of C–O, C–N, and C–C bonds, as well as the decomposition of the soy protein backbone [27]. When compared with the pure SM adhesive, the PHTO-modified adhesives showed a new peak at Stage II, which indicated that an unstable chemical bond (such as an ether bond) was formed in the adhesive system, and a different crosslinked structure was formed in the cured adhesive after using PHTO. Furthermore, a new shoulder peak appeared after Stage III, and the peak between 260–350 °C decreased when the PHTO concentration was increased from 5 to 20 wt %, which indicated that this new crosslinked structure had a better thermal stability [28].

**Scheme 1:**
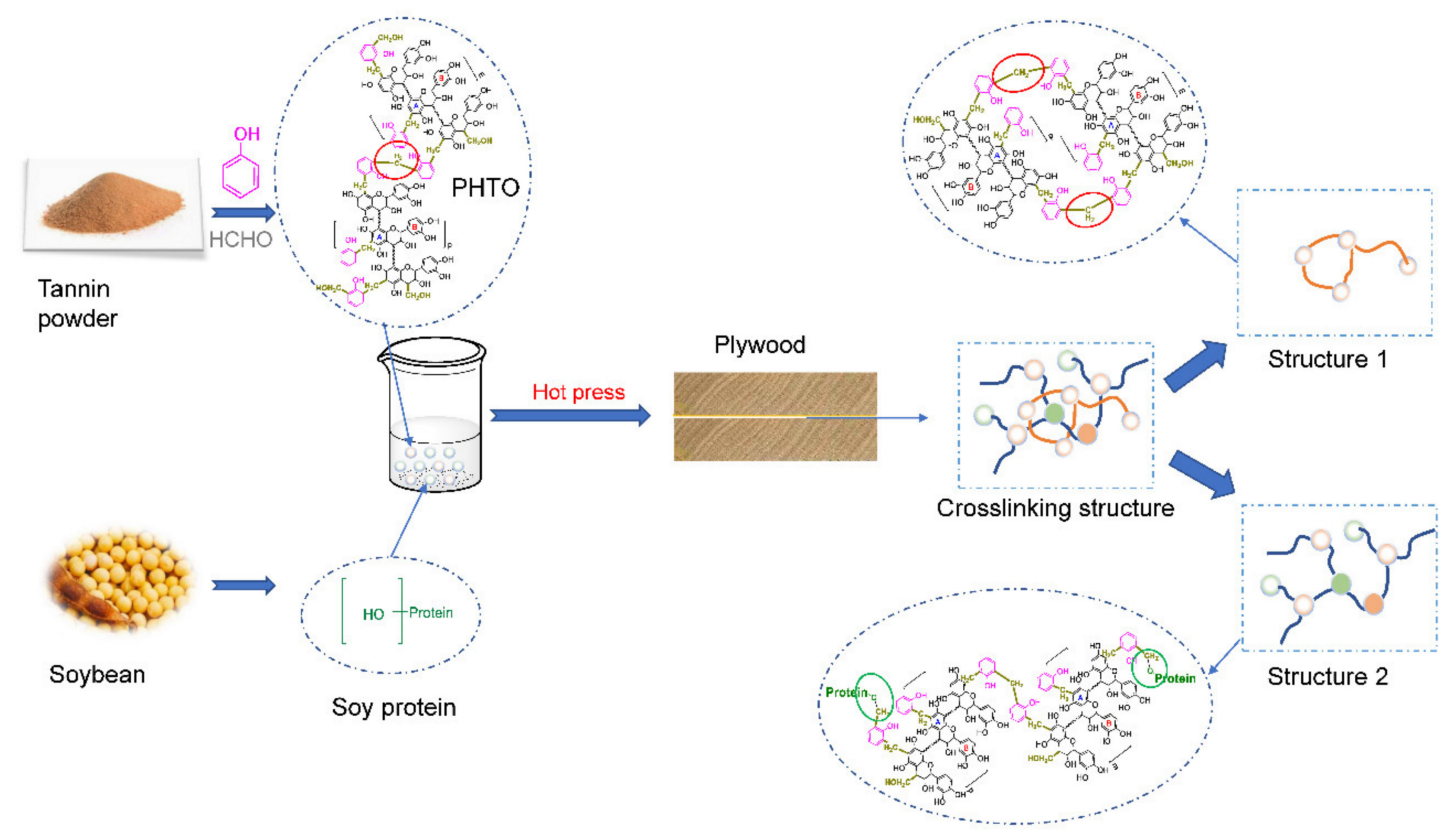
The reaction process of the PHTO and soy protein.

Figure S2 presents the FTIR spectra of the different adhesives. It was difficult to use the FTIR spectrum of the adhesive to indicate the chemical reaction between SM and LT since SM and LT have complex components. The addition of PHTOs resulted in an extensive broadening between 3250–3550 cm^−1^, indicating the existence of hydrogen bonds between the soy protein molecules and the PHTOs. When SPA forms a crosslinked network, the functional group vibration requires more energy, which manifests as a blue-shift in the FTIR spectrum [29]. As the PHTO concentration was increased from 0 to 20 wt %, amide I and II experienced a blue-shift from 1650 to 1658 cm^−1^ and from 1535 to 1541 cm^−1^, respectively. The blue shift in the PHTO-modified adhesive spectrum shows that the soy proteins formed denser structures than the pure SM adhesive. This indicates that a denser structure was formed due to the formation of the ether linkages between the hydroxyl groups of the protein molecules and the phenolic hydroxymethyl groups of PHTOs. The chemical linkages increased the extent of cross-linking within the adhesives and, subsequently, improved the water resistance of the modified SPAs, which was in accordance with Zhang’s research [30]. The reaction process between the soy protein molecules and the PHTO molecules is depicted in Scheme 1. During the curing of the adhesive, the hydroxymethyl group in PHTO reacted with a protein to form a network, while the PHTO underwent self-condensation polymerization in order to form another network. Combined with each other, these networks form an integrated crosslinked structure, which helped to improve the water resistance of the resultant adhesive. 

#### 3.2.2. Hydrolytic Stability

A residual rate test was used to determine the hydrolytic stability [31]. The residual rate of the cured adhesives initially increased, and then decreased with the continuous addition of PHTO, as shown in Table 2. The highest residual rate of the sample was obtained at 89.2% when using SPAs with 10 wt % PHTO, which was 11.6% higher than that of pure SM. The residual rate increase was mainly due to the formation of crosslinked structures via reactions between the PHTOs and soy protein. These reactions prevented moisture intrusion and enhanced the water resistance of the adhesive. Further increasing the addition of PHTO to 20 wt % resulted in a decrease in the residual rate, which was possibly due to the adverse impact of residual PHTO on the hydrolytic stability. 

#### 3.2.3. Rheology Properties

Adhesive viscosity significantly affects the handling of adhesives in their applications. Table 2 presents the measured viscosities of all the samples. The viscosity of the pure SM adhesive was 18,560 mPa·s, while the adhesive containing 5 wt % PHTO demonstrated a viscosity of 125,600 mPa·s, an increase of 577%. Further increasing the PHTO content, caused the adhesive viscosity to continuously increase. This increase occurred because the soy protein was in a loose and fluffy state under alkaline conditions, which increased the intermolecular entanglement, resulting in a high viscosity [6]. In addition, this increase was also attributed to an increase in hydrogen bonds and hydrophobic group interactions between the PHTOs and soy proteins [32].

#### 3.2.4. Solid Content Analysis

Solid content is a basic physical parameter of wood adhesive that influences the dimensional stability of the resultant plywood. Table 2 shows the solid content of the samples. A low solid content indicates that higher water content was introduced to the veneer stack. This water was removed in the hot press process, resulting in a long hot press time and poor dimensional stability of the resultant plywood. SPA with a low solid content also led to a lack of adhesive mass at an identical amount of adhesive spread, thus making it difficult to form an adhesive layer in plywood, which greatly reduced its bond stability [33]. However, the soy protein-based adhesive with a high solid content had high viscosity and it was difficult to distribute over the veneer surface. Furthermore, a higher solid content meant that more adhesive was present in the formulation, which increased its cost. Therefore, a solid content from 28% to 39% of soy protein-based adhesive is desired in the wood composites industry [34]. Pure SM adhesive exhibited the lowest solid content at 26.6%, as shown in Table 2. As the PHTO concentration increased from 5 to 20 wt % (mass ratios to the whole adhesive), the SPAs solid content gradually increased from 27.6% to 29.6% due to the high solid content in the PHTO (52.7%). When the addition of PHTO reached 10%, the solid content of the adhesive was 28.4%, which was comparable to the desired literature values.

#### 3.2.5. SEM Analysis and Moisture Absorption Measurement

**Figure 3:**
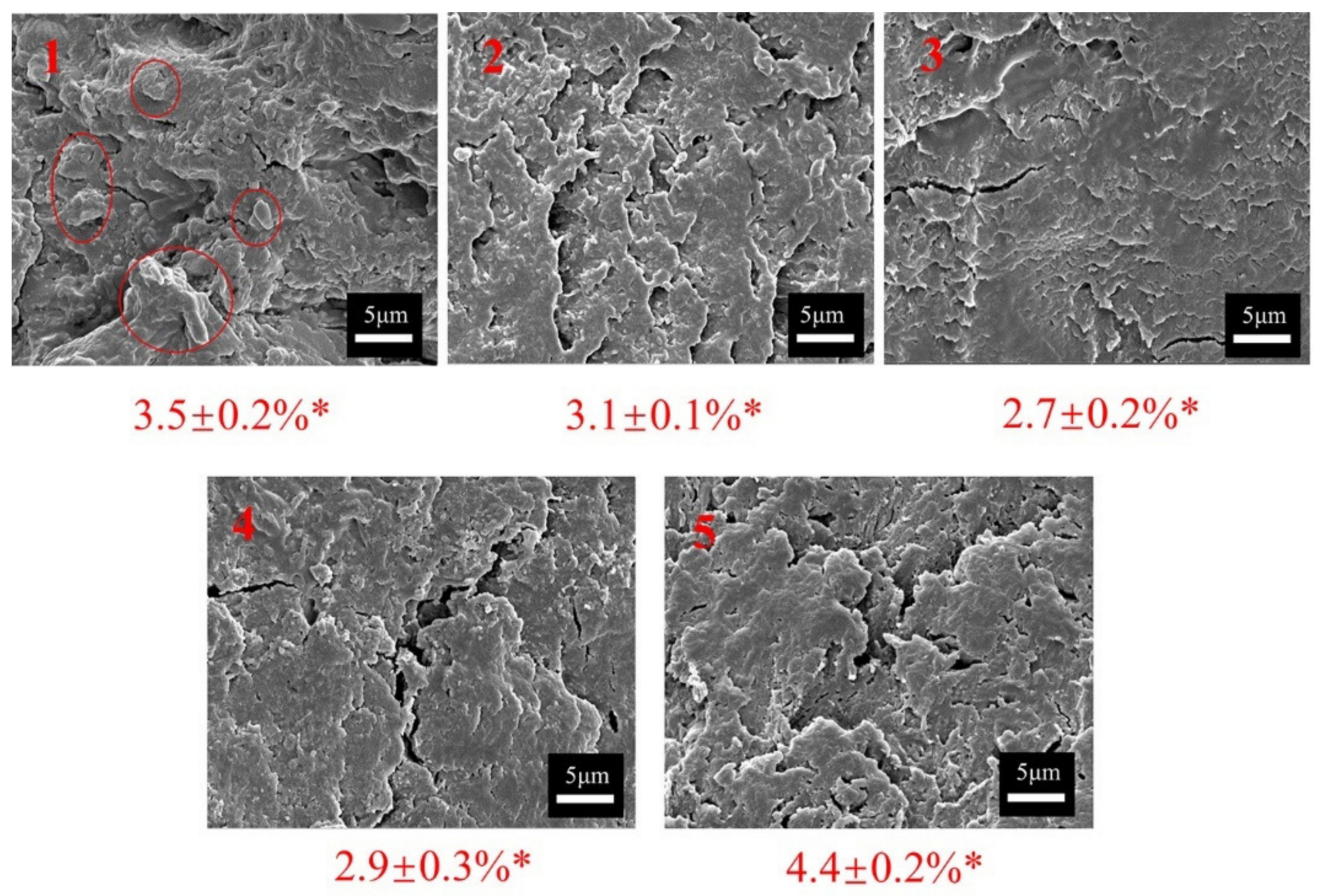
SEM images and moisture absorption measurements of the different adhesives: 1 (pure SM adhesive), 2 (5 wt % PHTO/SPA), 3 (10 wt % PHTO/SPA), 4 (15 wt % PHTO/SPA), and 5 (20 wt % PHTO/SPA), (* represents the moisture absorption value of different samples).

Figure 3 shows the fracture surfaces. The pure SM adhesive (adhesive 1) contained voids on the fracture surface and large protuberances in the red circles, which were attributed to the uneven adhesive system. This adhesive layer was easily broken by water, illustrating the adhesive’s water resistance. When the addition of PHTO reached 5 wt %, the fracture surface of adhesive 2 became smooth and compact. Further increasing the addition of PHTO to 10 wt %, the fracture surface of the adhesive samples became smoother and more compact with fewer protuberances due to the reaction between the PHTO and protein molecules which formed a denser crosslinked structure. As such, these experimental results are consistent with those of the FTIR spectra and the thermal stability analysis. As the PHTO addition increased to 20 wt %, adhesive 5 presented a disordered fracture surface again, which indicated that excessive amounts of PHTO caused its uneven distribution in the adhesive system, deteriorating its water resistance. 

**Figure 4:**
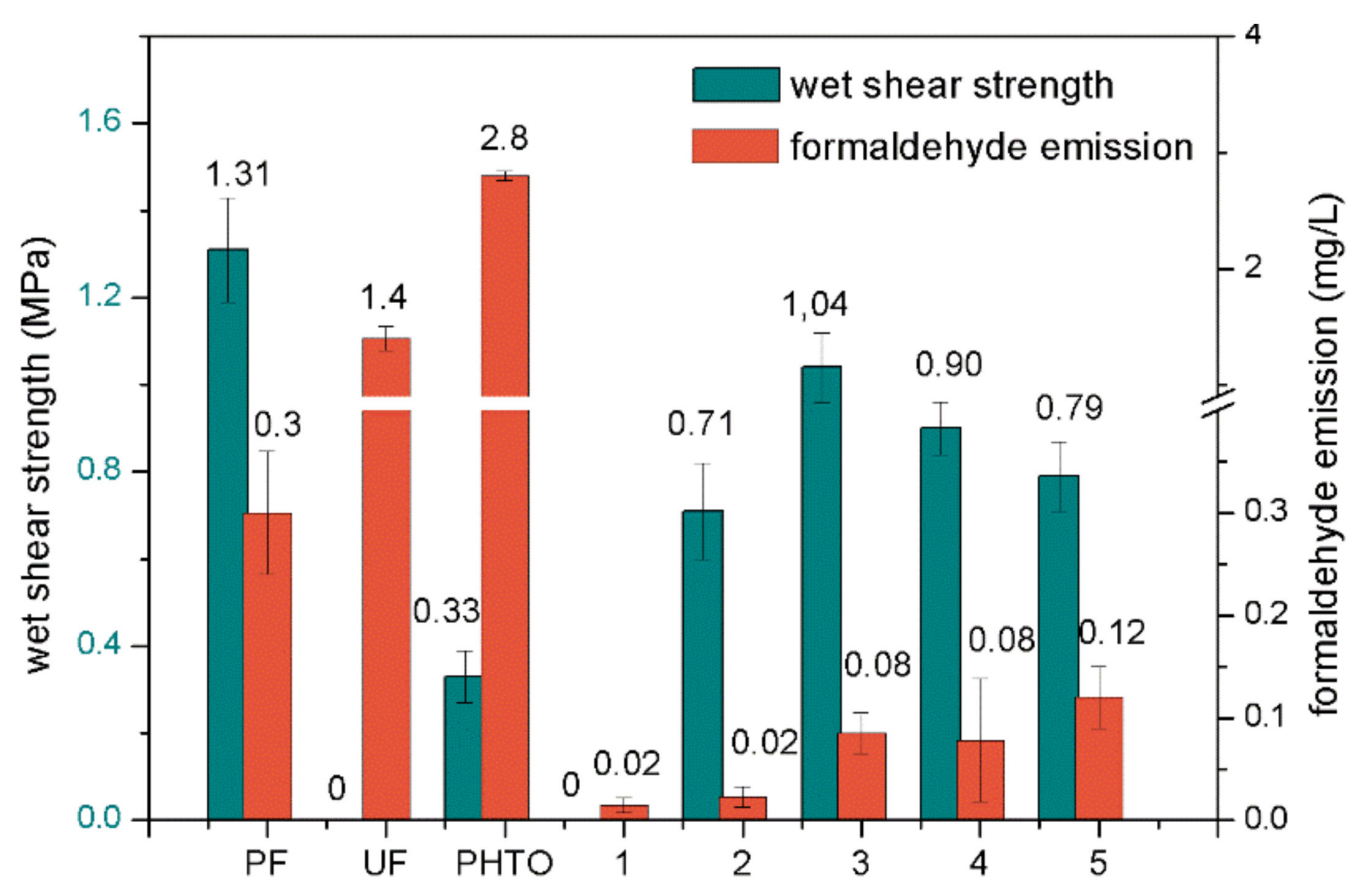
The wet shear strength and formaldehyde emission of plywood samples bonded with different adhesives (1 (pure SM adhesive), 2 (5 wt % PHTO/SPA), 3 (10 wt % PHTO/SPA), 4 (15 wt % PHTO/SPA), and 5 (20 wt % PHTO/SPA)).

Figure 3 shows the moisture absorption rates of cured adhesive samples. The moisture absorption rate of the pure SM adhesive was 3.5%. After gradually adding up to 10 wt % PHTO, the moisture absorption rate of the SPAs gradually decreased. This was attributed to the formation of a dense crosslinked structure due to the self-crosslinking reaction between the PHTOs and the reaction between the PHTOs and protein molecules. The moisture absorption rate increased by 63.0% as the PHTO content increased from 10 to 20 wt %. When only using PHTO as the adhesive, the water resistance and bonding performance of the adhesive were low (Figure 4). Excessive amounts of PHTO resulted in more PHTO remaining in the adhesive system after curing, which increased the moisture uptake of the system due to the presence of many hydrophilic groups. These results are also consistent with those of the FTIR spectra and the rheological properties analysis.

### 3.3. Performance Analysis of the Plywood Samples

#### 3.3.1. Wet Shear Strength Measurements

The wet shear strength of the three-ply plywood samples bonded with different adhesives was measured after being boiled in water at 100 °C for 3 h (Figure 4). Commercial PF and UF resins were used as the control. The sample with commercial PF resin reached 1.31 MPa, demonstrating its excellent wet shear strength. In contrast, the commercial UF resin and pure SM adhesive had no resistance to boiling water. The wet shear strength of the PHTO was only 0.33 MPa, which was far lower than the 1.33 MPa of the PF resin, which indicated that the PHTO was not a PF resin. This was because the amount of formaldehyde in the PHTO formulation was far lower than that of conventional tannin-PF resin. When 0 to 10 wt % PHTO was added to the SPAs, the wet shear strength increased from 0 to a maximum of 1.04 MPa, which met the requirement of exterior-use plywood according to the Chinese National Standard (GB/T 9846.3-2004). The maximum wet shear strength was the same level as the PF resin. The substantial improvement in the wet shear strength was due to (1) a higher crosslink density formed between the PHTO and protein molecules; and, (2) the self-crosslinked PHTO structure formed an integrated crosslinked structure by linking with protein molecules, which further increased the crosslink density [35]. However, further increasing the PHTO concentration beyond 10 wt % slightly decreased the shear strength. The wet shear strength decreased close to that of PHTO as more PHTO was added, which was attributed to the low water resistance of the PHTO itself, thus decreasing the water resistance of the adhesive system and the wet shear strength of the resultant plywood. These results are consistent with the results of the FTIR analysis, thermal stability analysis, rheology properties analysis, and moisture absorption measurements. 

#### 3.3.2. Formaldehyde Emission of Plywood

Figure 4 shows the formaldehyde emission values of the plywood samples. The formaldehyde emission of the commercial PF and UF resins were 0.3 and 1.4 mg/L, respectively. The formaldehyde emission of PHTO was 2.8 mg/L, which was far higher than that of the PF and UF resins, indicating that PHTO showed a much higher amount of free formaldehyde than PF or UF resins. The formaldehyde emission of the plywood bonded by pure SM adhesive was 0.02 mg/L. This formaldehyde emission must have been due to the degradation of a wood component since no formaldehyde was added to the SM adhesive. When the PHTO was increased from 5 to 20 wt %, the formaldehyde emission of the plywood bonded by the PHTO/SM adhesive increased slightly from 0.02 mg/L to a maximum of 0.12 mg/L. This value was significantly lower than the highest standard requirement for interior-use plywood (*E*_0_ ≤ 0.5 mg/L) (GB/T 9846.3-2004) and it is similar to the formaldehyde emission level of solid wood. PHTO-modified SPAs presented excellent water resistance and suitable viscosity. The glued product presented an exceptional wet shear strength and extremely low formaldehyde emission, demonstrating its high value for practical use in the wood industry.

## 4. Summary and Conclusions

Tannin was reacted with formaldehyde/phenol to form an oligomer (PHTO) with methylene and hydroxymethylated phenols. The addition of PHTO in SPA increased the residual rate, decreased the moisture absorption rate of the resultant adhesives, and effectively increased the wet shear strength of the resultant plywood. The results were optimized at 10 wt % PHTO (based on the weight of the soybean meal powder), because the residual rate and moisture absorption rate reached 89.2% and 2.7%. The wet shear strength and formaldehyde emission of the resultant plywood were 1.04 MPa and 0.08 mg/L, respectively, which both met the outdoor use plywood bond strength requirement and *E*_0_ level. These findings were attributed to (1) the increased crosslink density due to the reaction between PHTO and protein molecules, which improved the water resistance of PHTO-modified SPAs and the resultant plywood; (2) an optimal solid content and viscosity helped the adhesive to penetrate and form mechanical interlocks during plywood hot pressing; (3) the addition of PHTO increased the thermal stability of the cured adhesive; and, (4) the formation of an integrated crosslinked structure combined the linking of chains between the PHTO and protein molecules with the self-crosslinked network between the PHTOs. This research provides a practical option for developing plywood with high-water resistance that can potentially be used in exteriors.

## Figures and Tables

**Table 1:** The physical properties of phenol hydroxymethylated tannin oligomer (PHTO) sample.

Sample	Viscosity (mPa·s)	Solid Content	pH Value
PHTO	287,700 (±980) *^a^*	52.7% (±0.3%)	10.3 (±0.2)

*^a^* Standard deviation.

**Table 2:** The physical properties of the different adhesives.

Adhesives	pH	Residual Rate (%)	Viscosity (mPa·s)	Solid Content (%)
1	7.2 (0.1) *^a^*	79.9 (0.2)	18,560 (1250)	26.6 (0.2)
2	9.4 (0.2)	81.1 (0.1)	125,600 (23100)	27.6 (0.3)
3	9.7 (0.1)	89.2 (0.3)	297,800 (12400)	28.4 (0.1)
4	9.8 (0.1)	81.9 (0.2)	303,600 (9800)	28.9 (0.3)
5	10.1 (0.2)	71.7 (0.4)	783,400 (37600)	29.6 (0.1)

^a^ Standard deviation. Adhesive samples: 1 (pure SM adhesive), 2 (5 wt % PHTO/SPA), 3 (10 wt % PHTO/SPA), 4 (15 wt % PHTO/SPA), and 5 (20 wt % PHTO/SPA).

## Supplementary Materials

The following are available online at https://www.mdpi.com/2073-4360/12/4/758/s1, Figure S1: The TGA curves of larch tannin (LT) and phenolic hydroxylmethylated tannin oligomer (PHTO), Figure S2: FTIR spectra of the different adhesive samples: 1(SM adhesive), 2(5 wt% PHTO/SPA), 3(10 wt% PHTO/SPA), 4(15 wt% PHTO/SPA), and 5(20 wt% PHTO/SPA).
