# Supplementary material for: Soybean Meal-Based Wood Adhesive Enhanced by Phenol Hydroxymethylated Tannin Oligomer for Exterior Use
Source: Polymers (Basel). 2020 Mar 31;12(4):758. doi: 10.3390/polym12040758 (PMC7240477; doi:10.3390/polym12040758)
Supplement: Supplementary file 1 [file polymers-12-00758-s001.pdf]

Supporting information for

A soybean meal-based wood adhesive enhanced by phenol-hydroxylmethylated tannin oligomer for exterior-use

Mingsong Chen [1], Yi Zhang [1], Yue Li [1], Sheldon Q. Shi [2], Jianzhang Li [1], Qiang Gao [1,*], Hongwu Guo [1,*]

The thermal stability of larch tannin (LT) and phenolic hydroxylmethylated tannin oligomer (PHTO) were analyzed and the results are presented in Figure S1. With respect to the tannin curve, weight loss from 150 °C to 300 °C is mainly due to the thermal decomposition of tannins and the discharge of CO_2, CO, H_2, and CH_4 [1]. For PHTO, the amount of thermal weight loss was lower when compared to tannin. TGA measurements showed that phenol hydroxymethylation changed tannin's thermal properties and resulted in a more thermally stable PHTO structure. Differential Thermal Gravimetry (DTG) depicted two peaks on the tannin curve at 225 °C and 272 °C. These peaks merged to another two peaks, while new peaks appeared at between 300 °C and 500 °C. These results indicated that a new thermal-stable structure was generated in the PHTO.

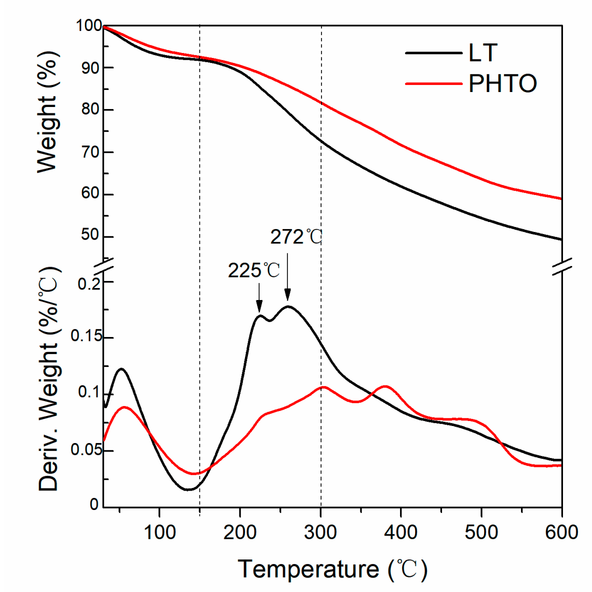

Figure S1. The TGA curves of larch tannin (LT) and phenolic hydroxylmethylated tannin oligomer (PHTO).

The FTIR spectra of the different adhesives are presented in Figure S2. Based on the results shown in Figure S2, the following absorption peaks were observed in all the tested adhesives: 1) O–H and N—H stretching vibrations from hydroxyl groups and amide groups (3,300-3,500 cm^{-1}); 2) C–H stretching vibrations from methyl groups (2,970 cm^{-1} and 2,930 cm^{-1}) and methylene groups (2,900 cm^{-1} and 2,870 cm^{-1}); 3) the stretching vibration of C=O (amide I, 1,650-1,668 cm^{-1}), deformation vibration of N–H (amide II, 1,535-1,547 cm^{-1}), and stretching vibration of C–N and N–H (amide III, 1,230-1,240 cm^{-1}); 4) the stretching vibration of COO– (1,390-1,410 cm^{-1}); and 5) the C–O stretching vibration (1,058 cm^{-1}) [2]. The addition of PHTOs resulted in an extensive broadening between 3,250-3,550 cm^{-1}, indicating the existing hydrogen bonding between soy protein molecules and PHTOs. When SPA was crosslinked and formed a network, the structure became dense and ordered. Thus, the vibration of functions needed more energy, which presented a blue shift in IR [3]. As the PHTO concentration was increased from 0 to 20 wt%, amide I and II experienced a blue shift from 1,650 to 1,658 cm^{-1} and 1,535 to 1,541 cm^{-1}, respectively. The blue shift in the PHTO-modified adhesive spectrum shows that soy protein inter-molecules formed denser structures than the pure SM adhesive.

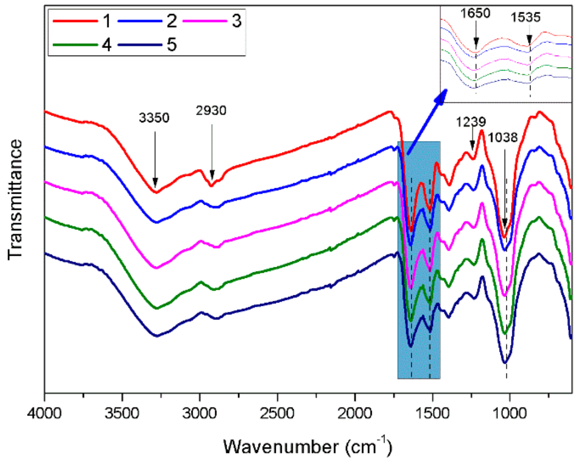

Figure S2. FTIR spectra of the different adhesive samples: 1(SM adhesive), 2(5 wt% PHTO/SPA), 3(10 wt% PHTO/SPA), 4(15 wt% PHTO/SPA), and 5(20 wt% PHTO/SPA).

References

1. Barbosa, V.; Ramires, E. C.; Razera, I. A. T.; Frollini, E. Biobased composites from tannin-phenolic polymers reinforced with coir fibers. *Ind. Crops Prod.* **2010**, *32*, 305–312.
2. Liu, H. J.; Li, C.; Sun, X.S. Soy-oil-based waterborne polyurethane improved wet strength of soy protein adhesives on wood. *Int. J. Adhes. Adhes.* **2017**, *73*, 66–74.
3. Yuan, C.; Chen, M.S.; Luo, J.; Li, X.N.; Gao, Q.; Li, J.Z. A novel water-based process produces eco-friendly bio-adhesive made from green cross-linked soybean soluble polysaccharide and soy protein. *Carbohyd. Polym.* **2017**, *169*, 417–425.
